# Development and optimization of a novel spectroscopic method to monitor crystallization behavior of BCS-II drug

**DOI:** 10.55730/1300-0527.3781

**Published:** 2025-11-22

**Authors:** Uditi HANDA, Anuj MALIK, Kumar GUARVE

**Affiliations:** 1 Guru Gobind Singh College of Pharmacy, Yamuna Nagar, Haryana, India; 2 Department of Pharmaceutics, MM College of Pharmacy, MM (DU), Mullana, Ambala, Haryana, India

**Keywords:** Supersaturation, crystallization kinetics, nucleation measurement, biopharmaceutical classification system (BCS)-II drug, UV absorbance graphical method, statistical analysis

## Abstract

This study investigated the crystallization behavior of dextromethorphan hydrobromide, a class II drug, to improve understanding of its supersaturation potential and formulation design. Conventional methods often lack the sensitivity or scalability needed for accurate detection of early nucleation events. To overcome these limitations, a novel process tracks real-time absorbance below turbidity levels to detect early molecular aggregation and phase separation. Crystallization kinetics of the drug was assessed at concentrations of 0.1, 0.2, and 0.3 mg/mL using three analytical techniques, namely, isothermal crystallization (baseline), ultraviolet (UV) absorbance (turbidity monitoring, UV-TM), and an optimized UV absorbance graphical method (UV-GM) (novel process). Induction times were recorded for each method, and statistical analysis was performed. In the descriptive statistics analysis, the one-sample test, and Pearson’s correlation were applied to evaluate consistency, variance, and the strength of association (R^2^ values) among the methods, thereby validating the reliability and precision of the developed UV-GM. As a result, at drug concentrations of 0.1, 0.2, and 0.3 mg/mL, nucleation induction times for isothermal crystallization were 80, 40, and 20 min; for UV-TM, 30, 20, and 5 min; and for UV-GM, 20, 20, and 10 min. The UV-GM demonstrated the highest precision with R^2^ values ranging from 0.8611 to 0.9439, compared to UV-TM (0.8223–0.9443) and isothermal crystallization (0.5444–0.6525), confirming itss superior reliability. Thus, it was concluded that the UV-GM offers a consistent, precise, cost-effective, and time-saving approach for estimating nucleation induction time. It also enables characterization of liquid–liquid phase separation, metastable zone width, and supersaturation potential, supporting rational formulation design and prediction of oral absorption in supersaturated systems.

## 1 Introduction

To improve the oral bioavailability of low water-soluble drugs, various solid forms are often used, including cocrystals, amorphous solid dispersions, and metastable polymorphic forms [1–2] When a supersaturated solid dissociates within a medium, the drug content typically attains a state of supersaturation, exceeding the thermodynamic solubility of its stable solid form. The concentration of the dissolved drug decreased over time, which was due to formation of precipitates. Simulating complex in vitro drug concentration patterns throughout the dissolution of supersaturated solid forms has proven to be highly challenging [3–5].

Molecular nucleation is a critical factor in solution crystallization, influencing various product characteristics such as the crystals appearance, polymorphic form, chiral form, and size distribution of the crystals produced [6]. The low supersaturation level is associated with the unseeded crystallization system due to the hypothetical nucleation process. Isothermal crystallization methods, ultraviolet (UV) absorbance (turbidity monitoring, UV–TM) can be utilized to represent the liquid–liquid phase segregation (LLPS) as well as the nucleation induction time and to estimate nucleation behavior [7–9]. The nuclei formation is approximately related with the interfacial energy of the crystallized substance [10,11], which is generally calculated using data regarding the induction time in the literature [12–14]. At a constant temperature, the time needed to generate and maintain the supersaturation state is defined as the induction time. Although determination of the induction time based on visual observation of crystal growth has been used previously [15–17], in recent years, nucleation onset has been monitored by measuring changes in the intensity of transmitted light in solution using turbidimetry instruments [16–21]; other methods, such as isothermal crystallization and UV-TMs, have also been developed to characterize nucleation profiles. These methods are based on variations in the induction time under equal supersaturation conditions [22–24]. A set of induction times measured under identical supersaturation and temperature conditions was used to determine the crystal nucleation rate. Despite these developments, comprehensive kinetic studies focusing on poorly water-soluble drugs in bulk form remain limited [25,26]. Prior research has emphasized the significance of isothermal crystallization in characterizing the crystallization behavior of polymers and active pharmaceutical ingredients [26–30].

The nucleation behavior of poorly water-soluble drugs, particularly BCS class II compounds like dextromethorphan hydrobromide (DXM), plays a critical role in determining their solubility enhancement and oral bioavailability. However, accurately evaluating nucleation kinetics remains challenging due to methodological limitations. Conventional techniques such as isothermal crystallization and UV-TMs provide limited insight, as they are either restricted to ambient conditions or require costly instrumentation and offer only partial data, such as the induction time or LLPS onset, and therefore lack the sensitivity to accurately detect early nucleation events [31–33]. To address these challenges, the present study focused on a comparative evaluation of three analytical approaches—namely, isothermal crystallization, UV-TM, and a novel UV-absorbance graphical method (UV-GM)—to assess nucleation kinetics over a comparable supersaturation range and monitor real-time absorbance variations at subturbidity levels, enabling the early detection of molecular aggregation and phase separation preceding visible crystal formation. This study aimed to identify the most effective and practical method for evaluating nucleation induction time and to elucidate the influence of solvent polarity on the crystallization kinetics of DXM using real-time UV absorbance analysis. It was hypothesized that increasing solvent polarity would decrease the nucleation rate by enhancing solvation and reducing molecular aggregation. The UV-GM, introduced and optimized herein, offers a cost-effective and comprehensive solution capable of simultaneously detecting the induction time, LLPS, metastable zone width (MSZW), and supersaturation potential. This comparative approach served to justify the adoption of the UV-GM as a superior alternative for rational formulation development and crystallization studies in supersaturation systems.

## 2 Materials and methods

### 2.1. Experimental material requirements

DXM (99.69% purity) was received as a sample gift (JRC Pvt Ltd., Gujarat). The organic solvent, N-N-dimethyl formamide (DMF), was analytical grade (99.5% purity; Nice Chemicals (P) Ltd., Kochi, Kerala, India). The disodium hydrogen phosphate (Na_2_HPO_4_) and potassium dihydrogen phosphate (KH_2_PO_4_) were of analytical grade at 99.2% and 99.5% purity, respectively (Merck Life Science Pvt. Ltd., Mumbai, India). Distilled water was used for preparation of the simulated intestinal fluid (SIP_sp_). The induction time was estimated using a UV-1800 dual beam UV-Visible (UV-Vis) spectrophotometer (Shimadzu Corp., Kyoto, Japan), and statistical analysis was performed using IBM SPSS Statistics for Windows 20.0 (IBM Corp., Armonk, NY, USA), with a 95% confidence interval (CI) applied throughout the study. The DXM concentrations (0.1, 0.2, and 0.3 mg/mL) were selected from preliminary solubility and supersaturation assessments to represent low, moderate, and high supersaturation levels, enabling measurable nucleation below the turbidity threshold for accurate absorbance detection.

### 2.2. Thermodynamic solubility of the DXM

Simulated intestinal fluid without pancreatin (SIF_sp_), containing DMF (1% v/v) to ensure complete dissolution of DXM due to its limited aqueous solubility, was employed as the test solvation medium, and all experiments were conducted at 37 °C unless otherwise specified. First, 10 mL of the test media was mixed with approximately 10 mg of DXM powder, and a magnetic stir bar was used to agitate the mixture continuously. The test solutions were filtered through 0.45-μm Whatman filter paper. The concentration of DXM in the filtrate was determined using a UV-Vis spectrophotometer (Shimadzu Corp.) at 278 nm. The concentration was monitored at 1-min intervals until a maximum peak value was observed, typically between 2 and 4 min after the DXM powder was added. This peak value was then used to calculate the thermodynamic solubility of the DXM. Subsequently, an abrupt decrease in concentration was observed due to DXM precipitation [33–35]. The thermodynamic solubility of DXM was assessed after 24 h of equilibration. All measurements were performed in triplicate.

### 2.3. Nucleation induction time measurement by the three different methods

#### 2.3.1. UV-GM (novel analytical method)

Supersaturation assay can be performed both with and without polymeric precipitate inhibitor and is used to investigate a polymer’s capacity to inhibit drug precipitation. Thus, optimization of the nucleation–induction time can also be used to describe the dynamics of crystallization. The induction time can be determined by UV spectroscopy based on changes in the absorbance of the drug solution [36–37]. A UV-absorbance graph was obtained to measure the induction time at 278 nm by detecting the sharp change in the absorption–time curve, which occurred due to the presence of nucleation and was quantified using Eq. (1).


(1) $$\text{CAb }(\text{cyst.})=({\text{Final}}_{(\text{Ass)}}\;\text{at T}1-{\text{Initial}}_{\text{(Ass)}}\;\text{at T}2)/2$$

**Figure 1:**
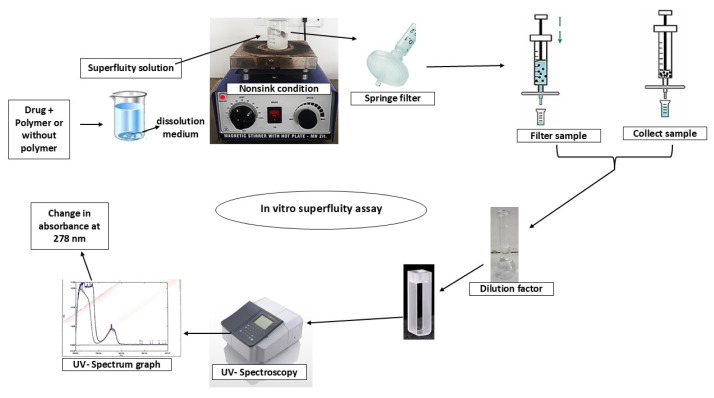
Schematic workflow of the novel UV absorbance graphical method (UV-GM) using the supersaturation assay.

Here, CAb (cyst.) denotes the change in absorbance due to crystallization at the induction time, Initial_(Ass)_ refers to the initial absorbance value at the supersaturated state, and Final_(Ass)_ is the final absorbance value at the supersaturated state over a time interval (T1 and T2) until sudden fluctuation of the absorbance takes place, which can be observed from a graph plotted as the change in absorbance (sudden highest fluctuation (nm)) versus time (min). The induction time was assessed by drawing straight lines from two distinct regions in the absorbance–time graph, and the point at which the two lines intersect was taken as the induction time [38]. The experiment was conducted in triplicate using the supersaturation assay in SIF_sp_ at 37 ± 0.5 °C, with samples withdrawal at 10-min intervals for up to 200 min, as illustrated in Figure 1. The UV-GM was calibrated and validated according to the previously established spectrophotometric protocol reported by Handa et al. [39], encompassing wavelength calibration, standard curve preparation, and validation of linearity, accuracy, and precision.

#### 2.3.2. Isothermal crystallization method

In the isothermal crystallization experiment, desupersaturation profiles of DXM under unseeded conditions were obtained [34]. Solvent shift was used to generate supersaturated DXM solutions, which were then continuously stirred to promote DXM crystallization. To clarify it briefly, 5 mg/mL of stock solution was obtained by dissolving DXM in DMF. Then, 50 mL of test medium was mixed with a portion of the stock solution to obtain DXM solutions of 0.1, 0.2, and 0.3 mg/mL. The experiment was performed under controlled isothermal conditions at 37 ± 0.5 °C to simulate physiological temperature. The supersaturation solutions were further filtered through 0.45-μm filter paper and stirred at 300 rpm in a beaker containing 50 mL of solution. Samples were collected at 10-min intervals for up to 200 min, and the DXM concentration was determined using the procedure outlined in the Section 2.2. Each preliminary DXM concentration in the desupersaturation profile was assessed in triplicate.

#### 2.3.3. UV-TM

The induction time is defined as the time required for a sudden increase in light intensity scattered from a drug solution upon the onset of nucleation. The nucleation induction time was used to determine crystallization onset. For DXM, light absorption was measured at 450 nm using a UV-Vis spectrophotometer. DXM itself does not absorb at this wavelength, making 450 nm ideal for detecting light scattering caused by nucleation rather than absorption. This allows precise identification of crystal formation [33,40,41]. Supersaturated drug solutions were prepared using the solvent-shift method at 37 ± 0.5 °C, where 0.5 mL of DMF solution containing 5 mg/mL of DXM was added to 50 mL of SIF_sp_ (without polymers) under continuous stirring at 300 rpm. Samples were collected at 10-min intervals for up to 200 min, and analyzed using a UV-Vis spectrophotometer to monitor crystallization.

### 2.4 Statistical analysis

Data were analyzed using IBM SPSS Statistics for Windows 20.0 (IBM Corp., Armonk, NY, USA) with a 95% CI. For the descriptive statistics, the one-sample t test and Pearson’s correlation were employed to assess consistency, variance, and the strength of association (R^2^ values) among the different analytical methods. These analyses validated the reliability, precision, and linear correlation of the developed UV-GM. All experiments were performed in triplicate (n = 3), ensuring reproducibility and sufficient statistical strength.

## 3 Results and discussion

### 3.1. Thermodynamic solubility of DXM

The thermodynamic solubility of DXM in the test medium was 0.005784 mg/mL at 37 °C in the DMF. This DXM value corresponds to the water-measured value and SIF_sp_ (0.0066 and 0.0079 mg/mL, respectively), and indicates that 1% DMF did not significantly influence the solubility of DXM in the test media. This was because the low DMF concentration ensured uniform mixing while minimizing any potential cosolvent effect on crystallization. Preliminary thermodynamic solubility studies confirmed that the presence of 1% DMF had a negligible impact on nucleation kinetics or overall crystallization behavior. Moreover, this small fraction acted only as a cosolvent to achieve uniform supersaturation without affecting nucleation kinetics or crystal morphology.

### 3.2. Three different methods (experimental drug precipitation profiles)

#### 3.2.1. Isothermal crystallization method

**Figure 2:**
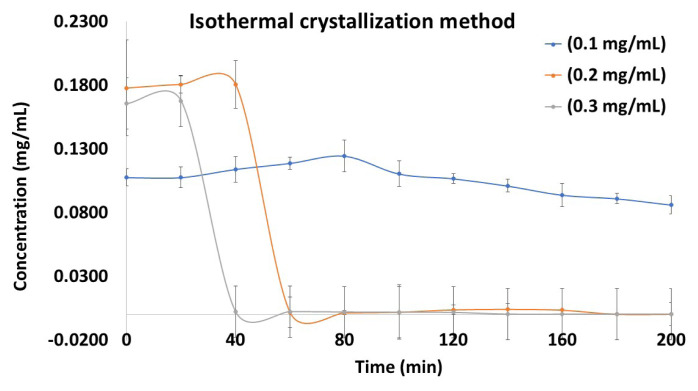
Nucleation induction time profiles of DXM using the isothermal crystallization method.

The desupersaturation profiles were acquired by performing the unseeded isothermal crystallization method. Figure 2 illustrates the correlation between the solvent polarity and crystallization rate, highlighting a significant decrease in induction time (faster nucleation) with increasing solvent polarity, emphasizing the impact of the solvent environment on crystallization kinetics. As a result, the initial concentrations were kept steady, and there was a sharp drop in concentration. They were used to estimate the induction time, defined as the duration of time necessary for nucleation to occur. When the initial concentrations were 0.3, 0.2, and 0.1 mg/mL, the induction times were approximately 20 ± 1.5, 40 ± 1.8, and 80 ± 2.3 min, respectively. The desupersaturation process began earlier with higher DXM content. The concentration of the drug was increased and the induction time decreased, which determined the precipitation of DXM, as presented in Table 1. The R^2^ value (regression coefficient) obtained from the graph plotted as the concentration versus time of the DXM supersaturation assay is presented in Table 2. It was observed that in the isothermal crystallization method, as the DXM content increased the R^2^ value improved. On the other hand, the variation in induction time was similar and stable when comparing each of the respective concentrations, i.e. 0.3, 0.2, and 0.1 mg/mL. However, it decreased in the concentration as the solution increased. Thus, nucleation and crystallization occurred faster at higher supersaturation levels.

#### 3.2.2. UV-TM

**Figure 3:**
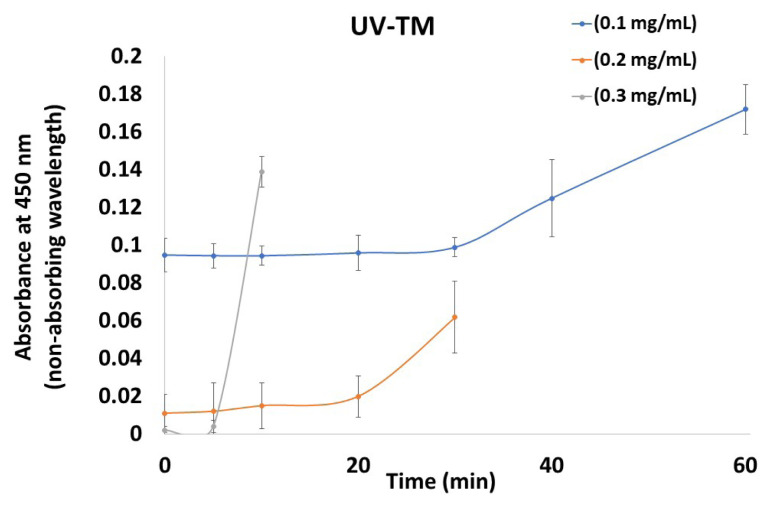
Nucleation induction time of DXM using UV absorbance (turbidity monitoring) (UV-TM).

The formation of LLPS was confirmed when UV light was scattered (due to the colloidal nature of the drug-rich phase) and was similarly observed at a non-absorbing wavelength, as shown in Figure 3. Each of the samples were examined with a UV-Vis spectrophotometer at 450 nm. The R^2^ value obtained from the graph plotted between the concentration and time in the DXM supersaturation assay is presented in Table 2. It was observed that in the UV-TM, as the concentration of DXM increased, the R^2^ value initially increased, and then slightly decreased. On the other hand, the variation in induction time was not equal when comparing each of the tested concentrations. However, it decreased as the concentration in the solution increased.

#### 3.2.3. UV-GM

**Figure 4:**
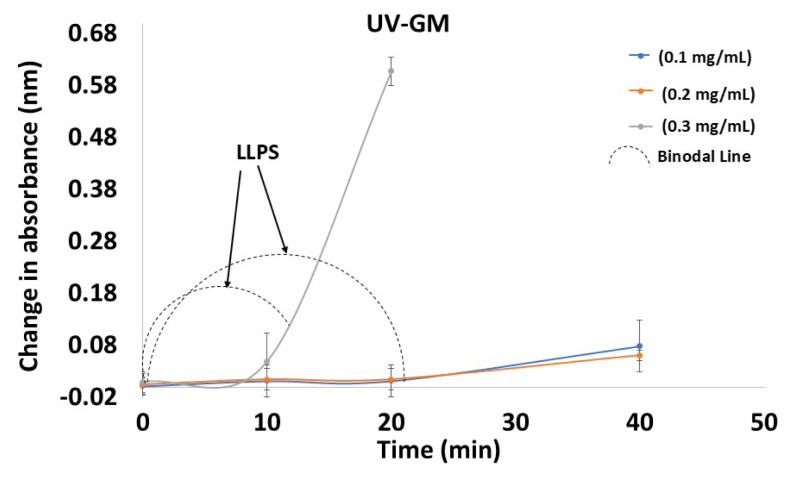
Nucleation induction time and LLPS profiles of DXM using the UV-GM.

The induction time was determined using the UV-GM with graphs plotted as absorbance versus time and by tracing the two distinct linear zones with regression lines. The induction time was determined by taking the cross-section endpoints of the regression lines for both points. Exemplary plots are displayed in Figure 4. The R^2^ value obtained from the graph plotted between the concentration and time in the DXM supersaturation assay is presented in Table 2. In the UV-TM, as the concentration of DXM increased the R^2^ value initially increased, and then slightly decreased. On the other hand, the variation in the induction time was uniform when comparing each of the tested concentrations. However, it decreased as the concentration in the solution increased. As shown in Table 1, the UV-GM exhibited consistently low variability (SD ≈ ± 1.0 – 1.1 min), indicating superior repeatability compared to the isothermal crystallization (SD ≈ ± 1.5 – 2.3 min) and UV-TM (SD ≈ ± 0.8 – 1.5 min). Furthermore, this method effectively distinguished small concentration changes (0.1 → 0.3 mg/mL), showing clear reductions in the induction time (20 → 10 min), confirming its higher sensitivity to concentration variations. Hence, the novel UV-GM provided not only a strong correlation (high R^2^) but also improved precision, reproducibility, and sensitivity compared to the other methods.

The decrease in induction time with increasing drug concentration can be attributed to the higher level of supersaturation achieved at higher concentrations, which enhances the frequency of molecular collisions and promotes faster nucleation. At higher supersaturation levels, the critical nucleus size decreases, facilitating more rapid formation of stable nuclei, thus shortening the induction time. This trend is consistent with classical nucleation theory and previous crystallization studies [22–24], confirming that nucleation kinetics are strongly dependent on the solute concentration and supersaturation level.

**Figure 5:**
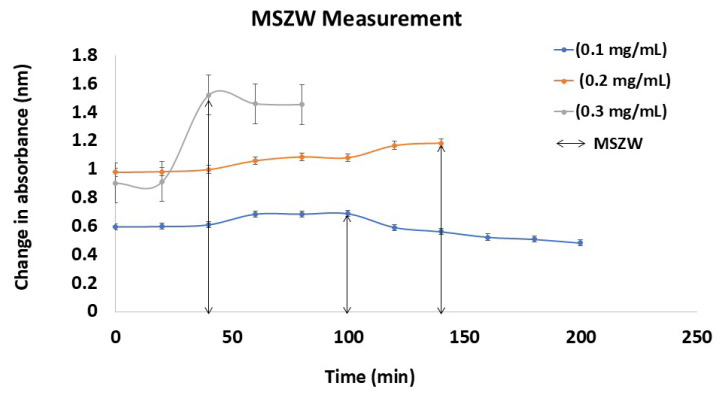
Absorbance profiles over time reflecting the supersaturation kinetics based on the MSZW measurement.

The binodal line marks the onset of LLPS, separating a homogeneous solution into drug-rich and drug-lean phases. In the absence of polymer, as the drug concentration decreases, the system remains below the binodal line, maintaining a stable single-phase solution. However, without polymer-mediated stabilization, the drug’s self-precipitation inhibition capacity diminishes, and at supersaturation thresholds near the binodal boundary, even minor fluctuations can lead to solid drug precipitation rather than LLPS, highlighting the role of polymers in extending kinetic stability and suppressing drug crystallization, as illustrated in Figure 4. This integrates binodal line behavior, the effects of the decreasing drug concentration, the absence of a polymer, and the tendency for drug self-precipitation. The MSZW is represented in Figure 5, where the MSZW predicts the supersaturation potential of the drug. The experimental drug precipitation profiles of DXM by isothermal crystallization, UV-GM, and UV-TM align with literature reports and confirm that in the absence of polymers, the decreasing drug concentration keeps the system below the binodal threshold, maintaining single-phase stability and preventing LLPS [42,43]. This supports the conclusion that lower drug concentrations lack sufficient supersaturation to drive phase segregation.

The findings of this investigation also identify parameters related to supersaturation behavior through the supersaturation test. The precipitation constants from the solution crystallization experiment reproduced the observed supersaturation behavior in a satisfactory manner (Figures 2–5). This indicates that the bulk solution was the region in which most of the crystal nucleation and growth took place throughout the DXM dissolving test.

The creation of a drug-rich phase (LLPS), which resembles tiny oil droplets, can occur between the spinodal breakdown of a supersaturated solution and highly supersaturated conditions. LLPS can also aid in the desupersaturation mechanism [44]. Crystallization may then occur from the drug-rich phase, resulting in solid-state formation. Only when the DXM concentration is higher than the solubility of amorphous DXM will the LLPS be observed [37]. However, it is necessary to scrutinize drug precipitation mediated by LLPS when higher supersaturation potential is produced by salt dissolution, amorphous solid dispersions, and cocrystals [33]. The current findings regarding the supersaturation behavior of DXM, assessed through supersaturation tests and precipitation constant measurements (Figures (2–4), are consistent with literature reports, which similarly describe LLPS and bulk solution crystallization as critical phenomena influencing nucleation, growth, and supersaturation maintenance during dissolution processes [42,44].

### 3.3. Statistical outcomes

Significant associations between various quantitative analyses of induction time and different methods were estimated using the one-sample t test. p > 0.05 indicated retention of the null hypothesis as the clear numerical differences in induction times, as shown in Table 3. No significant differences in the induction time were observed among the different methods across the increasing drug concentrations (Table 1), suggesting that the novel UV-GM is comparable to conventional techniques and was successfully optimized. This implies that the novel method behaves similar to conventional methods across different concentrations, which supports the conclusion that the novel method is reliable and comparable.

Although the R^2^ values of the UV-TM and UV-GM were comparable, indicating similar correlations between the concentration and induction time, the superiority of the proposed method extends beyond this parameter. Notably, it demonstrated higher sensitivity, as it was capable of detecting subtle variations in absorbance corresponding to early-stage nucleation events that were not discernible by the conventional UV-TM. This limitation arose because the UV-TM was measured at an absorbance wavelength (450 nm) that does not coincide with the λmax of DXM, whereas the proposed UV-GM detects at λmax (278 nm), providing stronger signals and enabling earlier detection of crystallization onset. Furthermore, the UV-GM showed better precision and repeatability, evidenced by lower standard deviations across all the tested concentrations (SD = ±1.0–1.1 min) compared to the UV-TM (SD = ±1.2–1.5 min). Additionally, enhanced baseline stability and data resolution minimized background noise, allowing more accurate identification of induction points and confirming its greater analytical reliability and sensitivity in crystallization monitoring. Thus, the UV-GM is also an optimized method like the other two methods. The UV-GM also serves as a time-efficient and cost-effective approach, providing informative data for crystallization studies, and enabling rational polymer selection for improved efficacy, as the the nucleation induction time can be detected simultaneously during the kinetic crystallization profile, in contrast to both the other methodologies. The methods provided in this research are useful for rationale formulation design and precise estimation of drug oral absorption in supersaturation-based drug delivery systems [37]. However, existing methods exhibit several methodological limitations. The isothermal crystallization method is restricted to ambient temperature conditions and is therefore not suitable for biorelevant media or for the simultaneous assessment of LLPS and the supersaturation index However, existing methods have some methodological limitations. The isothermal crystallization method is restricted to ambient temperature conditions and is therefore not suitable for biorelevant media or for assessment of LLPS and the supersaturation index. In addition, the UV-TM is limited by its reliance on a UV-Vis spectrometer equipped with an SI Photonics UV fiberoptic dip probe, which substantially increases experimental costs, because the probe alone costs approximately 2–3 lakh [33]. Without using this probe, the experimental variability increased, resulting in inaccurate results and limiting optimization. The developed UV-GM demonstrated enhanced sensitivity in detecting early-stage nucleation and molecular aggregation below turbidity levels. This aligns with recent spectroscopic and electrochemical investigations that emphasize the importance of real-time signal monitoring for improved analytical precision and molecular interaction profiling. These studies collectively report that integrating spectroscopic/electrochemical approaches enhances detection accuracy of subtle physicochemical transitions [45–48], supporting the reliability of absorbance-based kinetic monitoring in complex systems. Increasing solvent polarity was found to slow crystallization rates, consistent with our hypothesis, and was likely due to stronger solvation and reduced molecular aggregation. Such mechanistic insights align with prior studies in which the solute–solvent interaction strength has been shown to correlate inversely with ease of nucleation [49]. The method effectively captured these early changes, providing mechanistic insight and a reliable approach for evaluating nucleation kinetics, supporting optimization of crystallization processes in pharmaceutical systems. In accordance, our UV-GM exhibited superior reproducibility compared to conventional techniques, confirming its capability to characterize nucleation kinetics with high precision and cost-effectiveness. The cost-effectiveness of the proposed UV-absorbance method stems from its use of a standard UV-Vis spectrophotometer, minimal sample volume, and simplified procedure, eliminating the need for advanced instrumentation or complex preparation, while concurrently enabling detection of the supersaturation potential, LLPS, MSZW, and nucleation induction time capabilities not achievable with conventional methods.

## Conclusion

This study demonstrated that isothermal crystallization, UV-TM, and the novel UV-GM effectively estimate nucleation and precipitation kinetics of DXM across varying concentrations (0.1–0.3 mg/mL). The results revealed a concentration-dependent decrease in the nucleation induction time across all the methods, with values for isothermal crystallization ranging from 80 to 20 min, for UV-TM from 30 to 5 min, and for UV-GM from 20 to 10 min, confirming faster crystallization with higher supersaturation. The UV-GM exhibited the most consistent performance and highest R^2^ values (0.8611–0.9439), and was statistically validated using the one-sample t test (SPSS 20.0, 95% CI). This novel approach effectively characterized DXM supersaturation, supporting formulation design and oral absorption prediction in supersaturation dosage forms. This cost-effective, efficient method allows precise estimation of crystallization parameters like the nucleation time, LLPS, MSZW, and supersaturation. Ongoing studies aim to deepen our understanding of supersaturation in diverse BCS-II drugs. The UV-GM showed enhanced sensitivity for early-stage nucleation, but potential interference from impurities and detection limits may affect accuracy. Future work should validate the method across varied impurity profiles, and employ complementary techniques to further improve reliability and applicability. Additionally, the effects of mixed solvent systems on DXM crystallization kinetics should be investigated, by applying the UV-GM. Such studies would further support its broader application in formulation development and control of crystallization processes in pharmaceutical systems.

## Figures and Tables

**Table 1:** Estimation of the nucleation induction time of DXM by the different methods.

Drug precipitation profiles Methods	Parameter (min)	Concentration (mg/mL)		
		0.1	0.2	0.3
Isothermal crystallization	Induction time	80 ± 2.3	40 ± 1.8	20 ± 1.5
UV-TM	Induction time	30 ± 1.5	20 ± 1.2	5 ± 0.8
UV-GM	Induction time	20 ± 1.1	20 ± 1.0	10 ± 0.9

**Table 2:** The R^2^ values by the different methods.

Drug precipitation profiles Methods			Isothermal crystallization	UV-TM	UV-GM
R^2^ value	Concentration (mg/mL)	0.1	0.5444	0.8253	0.9092
		0.2	0.5734	0.9443	0.9439
		0.3	0.6525	0.8223	0.8611

**Table 3:** Estimation of the induction time by the different methods using the one-sample test (SPSS).

Induction time	Test value = 3					
Drug concentration (mg/mL)	T	Df	Sig. (2-tailed)	Mean difference	95% CI of the difference	
					Lower	Upper
(0.1)	2.173	2	0.162	40.333	−39.52	120.19
(0.2)	3.550	2	0.071	23.667	−5.02	52.35
(0.3)	1.965	2	0.188	8.667	−10.31	27.64
